# Supplementary material for: Metabolic Reprogramming of Urothelial Carcinoma—A Theragnostic Target for Betulinic Acid
Source: Int J Mol Sci. 2025 Jun 11;26(12):5598. doi: 10.3390/ijms26125598 (PMC12192694; doi:10.3390/ijms26125598)
Supplement: Supplementary file 1 [file ijms-26-05598-s001.zip › ijms-3633957-supplementary.pdf]

Supplementary Information

Figure S1

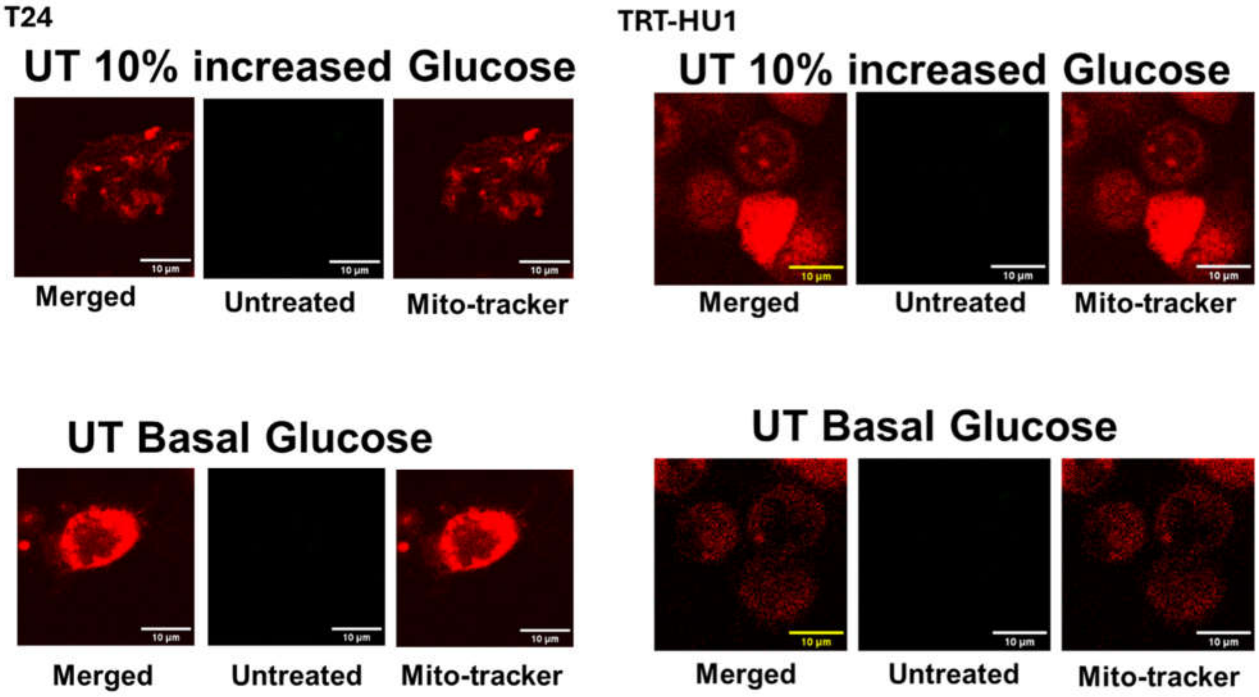

Figure S1. Confocal images depicting MitoTracker red fluorescence in T24 and TRT-HU1cells left untreated (UT) with BA-FITC at basal glucose (bottom panels) and 10% increased glucose (top panel). MitoTracker red fluorescence decreased in T24 cells with 10% increased glucose relative to basal glucose whereas the increase in MitoTracker red fluorescence of TRT-HU1 cell grown at 10% increased glucose relative to basal glucose implies that glucose toxicity inflict mitochondrial membrane disruptions evoked by glucose scarcity in T24 cell at basal glucose.

Figure S2

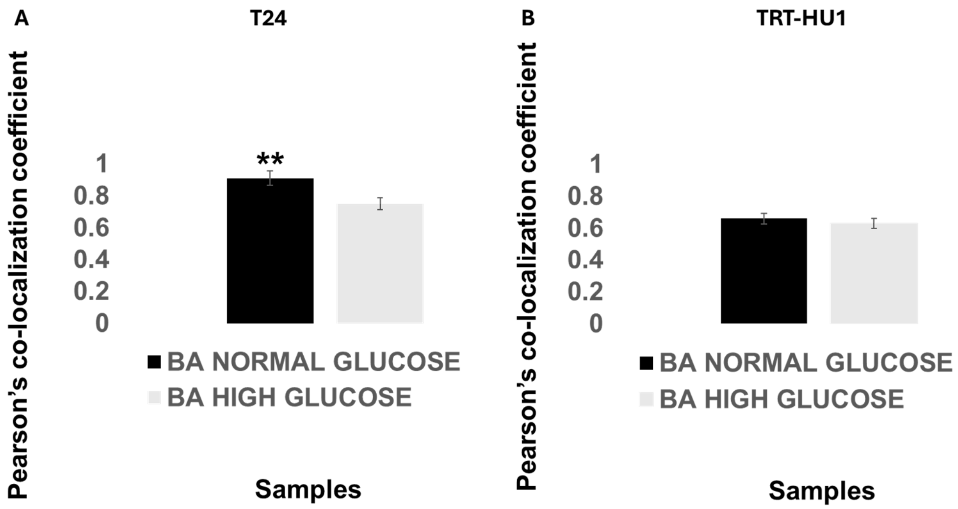

Figure S2. Pearson's co-localization coefficient in (A) T24 and (B) TRT-HU1cells. (A) Pearson's correlation coefficient for fluorescence colocalization in T24 declined significantly from 0.91 at normal glucose(basal, displayed by dark bars) to 0.75 at 10% increased glucose(high glucose, displayed by grey bars), ***p* value $\leqslant$ 0.01. (B) Pearson's correlation coefficient for fluorescence colocalization in TRT-HUI decreased insignificantly from 0.66 at normal glucose(basal, displayed by dark bars) to 0.63 with a 10% increase in glucose (high glucose, displayed by grey bars)

Experimental Procedures for the preparation of [BA-FITC]:

Synthesis of tert-butyl (3-aminopropyl) carbamate:

To a stirred solution of 1,3-diaminopropane (2.0 g, 0.026 mol) in 15mL anhydrous ACN, (BoC)2O (3.71 mL, 0.0160 mol) in 5 mL ACN was added dropwise at 0°C under nitrogen atmosphere. The reaction mixture was warmed to room temperature and stirred for 24 h. The solvent was removed under reduced pressure and the thick oil so obtained was diluted with CH2Cl2. The organic layer was washed with brine (3 × 20 mL), dried over anhydrous Na2SO4, and concentrated under reduced pressure to give product as gummy oil. The crude oily substance was then purified through a filtering column, which was used for the next step. 1H NMR (300 MHz, CDCl3): δ 4.94 (br s, 1H), δ 3.18 (d, J=6.0Hz, 2H), δ 2.76-2.71 (m, 2H), δ 1.63-1.54 (m, 2H), δ 1.41 (s, 9H), δ 1.36 (br s, 2H). [Ref. J. Med. Chem. 2014, 57, 4263-4272.]

Synthesis of tert-butyl (3-((1R,3aS,5aR,5bR,7aR,9S,11aR,11bR,13aR,13bR)-9-hydroxy-

5a,5b,8,8,11a-pentamethyl-1-(prop-1-en-2-yl)icosahydro-1H-cyclopenta[a]chrysene-3a-carboxamido)propyl)carbamate [BA (NH-Boc)]:

To a solution of betulinic acid (100 mg, 0.218 mmol) in dry DCM, NH-Boc compound (76 mg, 0.436 mmol), NEt3 (120µL, 0.32757 mmol), DMAP (10 mol %), EDC (251 mg, 1.309 mmol) and HOBt (176 mg, 1.302 mmol) were sequentially added. The reaction mixture were kept stirring overnight at rt. After extraction in EtOAc the solvent was removed under vaccum. The crude product was isolated by flash column chromatography on (100-200 mesh) silica gel with (95:5) DCM/MeOH as an eluent to obtain the pure product [BA (NH-Boc)] [Rf ≈ 0.5, 100mg, 90%].

1H NMR (300 MHz, CDCl3):

δ 6.33 (br s, 1H), δ 4.90 (br s, 1H), δ 4.74 (s, 1H), δ 4.58 (s, 1H), δ 3.39-3.11 (m, 5H), δ 2.50-2.40 (m, 1H), δ 2.04-1.87 (m, 2H), δ 1.80-1.71 (m, 2H), δ 1.68 (s, 3H), δ 1.63-1.47 (m, 9H), δ 1.44 (s, 9H), δ 1.41-1.30 (m, 4H), δ 1.25 (s, 6H), δ 1.17-1.14 (m, 1H), δ 0.96 (s, 3H), δ 0.95 (s, 3H), δ 0.92 (s, 3H), δ 0.89-0.85 (m, 2H), δ 0.81 (s, 3H), δ 0.74 (s, 3H), δ 0.67 (d, J=9.0Hz, 1H). (ESI-MS): Calcd for C38H65N2O4 (M+H)+ : 614, found 614, (M-56)+: 558, (M-100)+: 514

Synthesis of (1R,3aS,5aR,5bR,7aR,9S,11aR,11bR,13aR,13bR)-N-(3-aminopropyl)-9-hydroxy-5a,5b,8,8,11a-pentamethyl-1-(prop-1-en-2-yl)icosahydro-1H-cyclopenta[a] chrysene-3a-carboxamide [BA (NH2)]:

Compound [BA (NH-Boc)] (50 mg, 0.0815 mmol) diluted in DCM was treated with (4M) Dioxane.HCL at 0°C. After the addition was complete, the cooling bath was removed and the mixture stirred at rt for 18h. Then the solvent was removed under reduced pressure and the crude mixture thus obtained was washed with pentane /ether (1:1) used for the next step without further purification. (ESI-MS): Calcd for C33H57N2O2

(M+H)+ : 513.4420, found 514.

Synthesis of (1R,3aS,5aR,5bR,7aR,9S,11aR,11bR,13aR,13bR)-N-(3-(3-(3',6'-dihydroxy-3-oxo-3H-spiro[isobenzofuran-1,9'-xanthen]-5-yl)thioureido)propyl)-9-hydroxy-5a,5b,8,8,11a-pentamethyl-1-(prop-1-en-2-yl)icosahydro-1H-cyclopenta[a]chrysene-3a-carbo xamide [BetA-FITC]:

To a solution of [BA (NH2)] (10 mg, 0.0194 mmol) in dry DCM, FITC (8.0 mg, 0.0205 mmol) and NEt3 (6 µL) were sequentially added. The reaction mixture was kept stirring overnight at rt under dark condition. After extraction in EtOAc and solvent removal, the crude

product was purified by flash column chromatography on neutral alumina with (9:1) DCM/MeOH as an eluent [Rf ≈ 0.4, 10.0 mg, 53%]. 1H NMR (500 MHz, DMSO-d6): δ 10.15 (s, 2H), δ 8.35 (br s, 1H), δ 7.80 (br s, 1H), δ 7.16 (d, J=8.0Hz, 1H), δ 6.69 (s, 2H), δ 6.57 (dd, J= 15.0Hz, J=7.5Hz, 5H), δ 4.70 (m, 1H), δ 4.30-4.26 (m, 1H), δ 4.11-4.05 (m, 1H), δ 3.62-3.50 (m, 3H), δ 3.16 (d, J=3.0Hz, 2H), δ 3.14-3.11 (m, 1H), δ 2.99-2.96 (m, 1H), δ 1.82-1.56 (m, 12H), δ 1.45-1.14 (m, 15H), δ 1.08-0.65 (m, 20H). (ESI-MS): Calcd for $C_{54}H_{68}N_3O_7S$ $(M+H)^+$: 903.4778, found 906.0

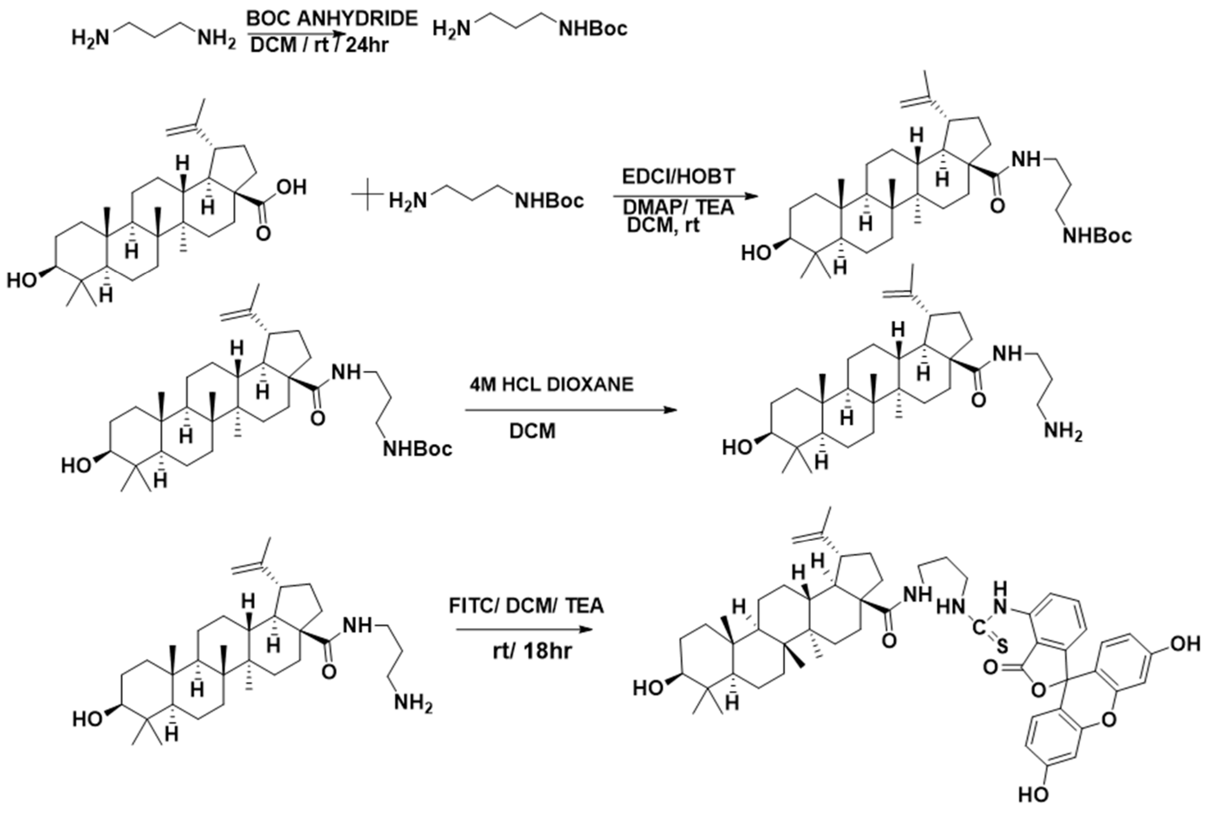

Figure S3 Schematic representation of preparation of BA-FITC

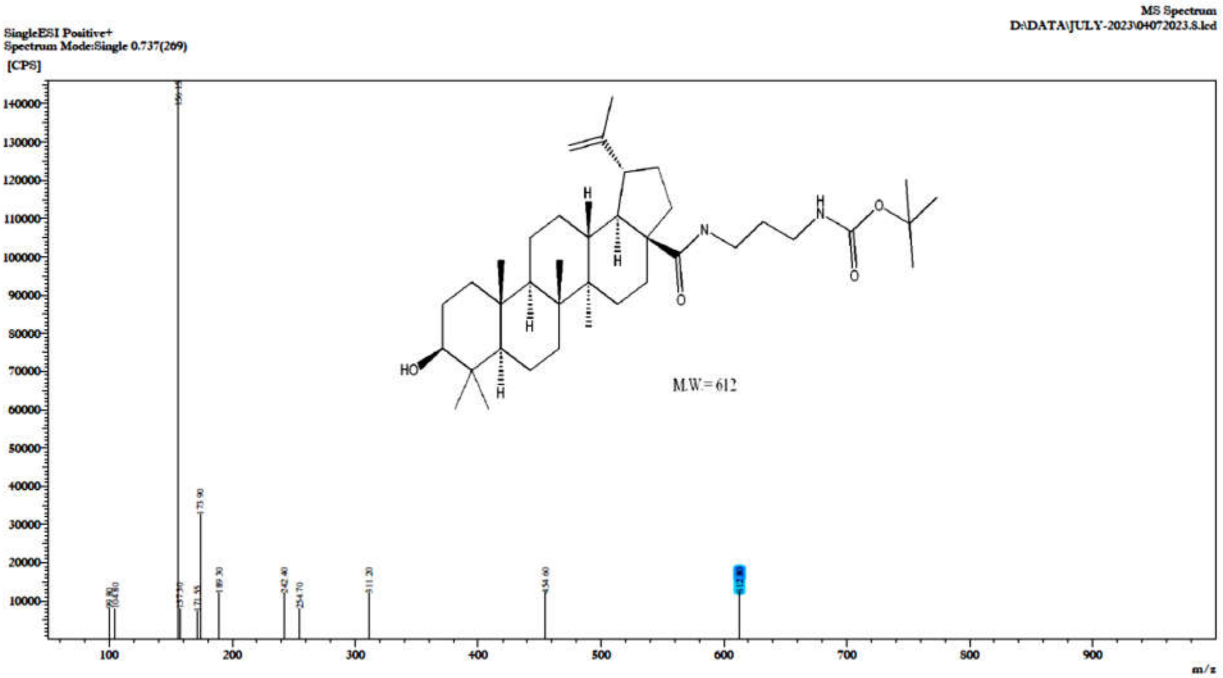

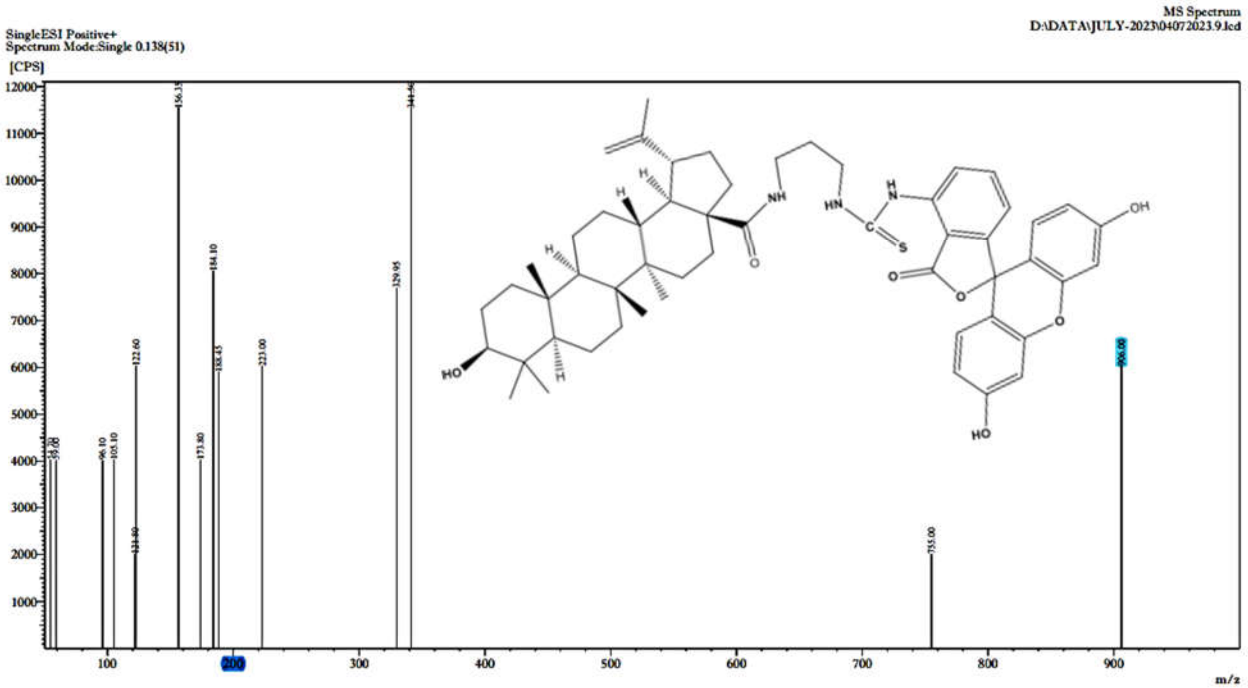

Figure S4. Mass spectrum of an intermediate (top) and of BA-FITC (bottom) with molecular structure and molecular weight of 612 and 903 Daltons, respectively. Key peaks are highlighted in blue.

Figure S5

A

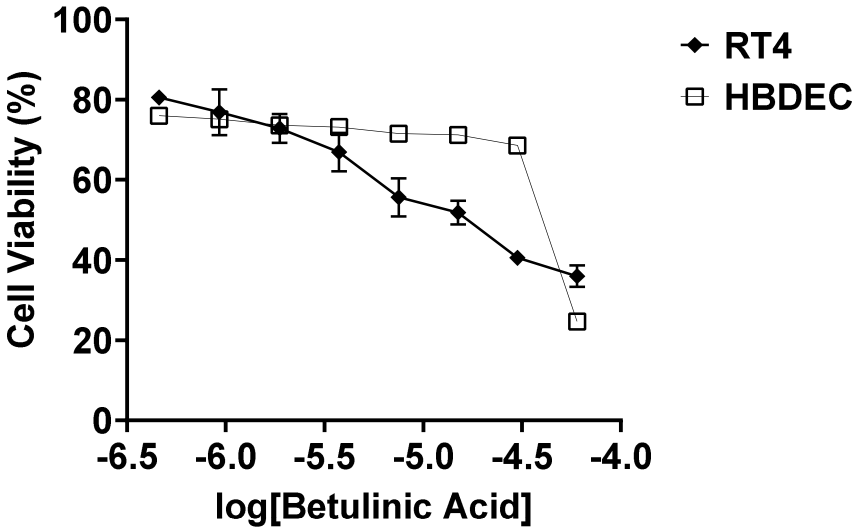

B

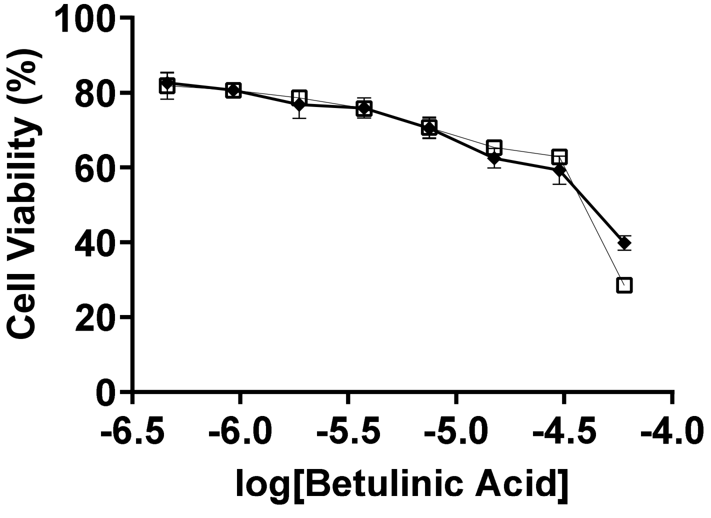

Figure S5. Betulinic Acid (BA) induced cytotoxicity at basal glucose (A) and 10% increased glucose (B) in urothelial carcinoma cell line RT4 and normal urothelial cell line HBDEC

Figure S6

A

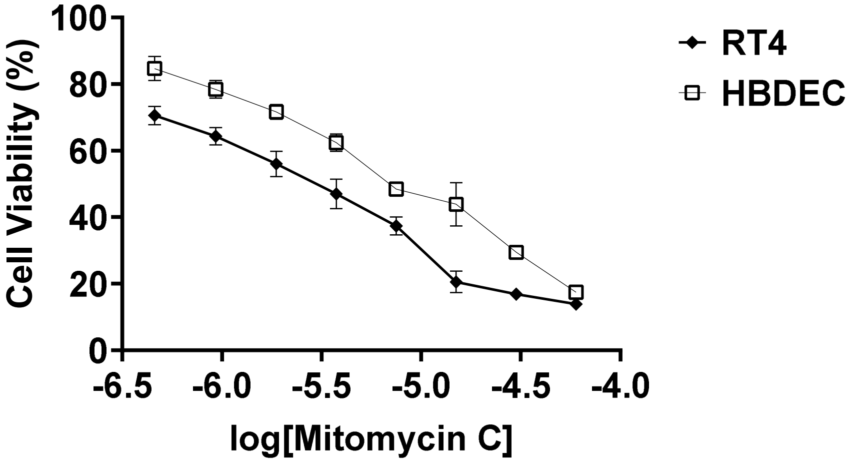

B

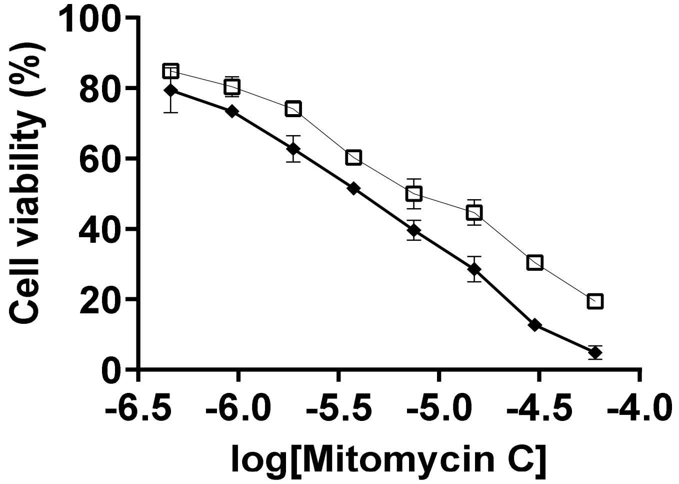

Figure S6. Mitomycin C (MC) induced cytotoxicity at basal glucose (A) and 10% increased glucose (B) in urothelial carcinoma cell line RT4 and normal urothelial cell line HBDEC

Figure S7

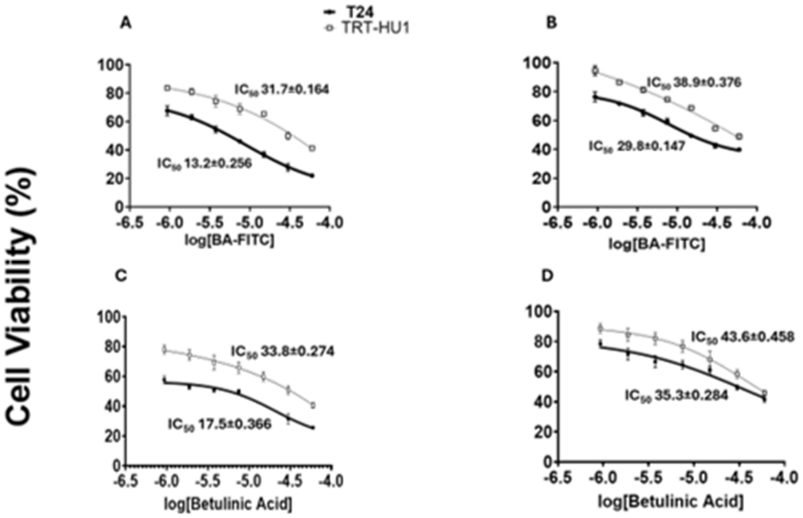

Figure S7. Normalized viability of T24 and TRT-HU1 cells plotted relative to the viability at the lowest dose of BA FITC(Panel A-B) and Betulinic Acid (Panel C-D) at basal glucose (left panels) and 10% increased glucose (right panels), respectively
